# Neurosurgical Team Acceptability of Brain–Computer Interfaces: A Two-Stage International Cross-Sectional Survey

**DOI:** 10.1016/j.wneu.2022.05.062

**Published:** 2022-08

**Authors:** Simon C. Williams, Hugo Layard Horsfall, Jonathan P. Funnell, John G. Hanrahan, Andreas T. Schaefer, William Muirhead, Hani J. Marcus

**Affiliations:** 1 Victor Horsley Department of Neurosurgery, National Hospital for Neurology and Neurosurgery, London, United Kingdom; 2 Wellcome/EPSRC Centre for Interventional and Surgical Sciences (WEISS), London, United Kingdom; 3 The Francis Crick Institute, Sensory Circuits and Neurotechnology Laboratory, London, United Kingdom; 4 Department of Neuroscience, Physiology & Pharmacology, University College London, London, United Kingdom

**Keywords:** Acceptability, BCI, BMI, Brain–computer interface, Brain–machine interface, Clinician, Neurosurgical team, Survey

## Abstract

**Objective:**

Invasive brain–computer interfaces (BCIs) require neurosurgical implantation, which confers a range of risks. Despite this situation, no studies have assessed the acceptability of invasive BCIs among the neurosurgical team. This study aims to establish baseline knowledge of BCIs within the neurosurgical team and identify attitudes toward different applications of invasive BCI.

**Methods:**

A 2-stage cross-sectional international survey of the neurosurgical team (neurosurgeons, anesthetists, and operating room nurses) was conducted. Results from the first, qualitative, survey were used to guide the second-stage quantitative survey, which assessed acceptability of invasive BCI applications. Five-part Likert scales were used to collect quantitative data. Surveys were distributed internationally via social media and collaborators.

**Results:**

A total of 108 qualitative responses were collected. Themes included the promise of BCIs positively affecting disease targets, concerns regarding stability, and an overall positive emotional reaction to BCI technology. The quantitative survey generated 538 responses from 32 countries. Baseline knowledge of BCI technology was poor, with 9% claiming to have a good or expert knowledge of BCIs. Acceptability of invasive BCI for rehabilitative purposes was >80%. Invasive BCI for augmentation in healthy populations divided opinion.

**Conclusions:**

The neurosurgical team's view of the acceptability of invasive BCI was divided across a range of indications. Some applications (e.g., stroke rehabilitation) were viewed as more appropriate than other applications (e.g., augmentation for military use). This range in views highlights the need for stakeholder consultation on acceptable use cases along with regulation and guidance to govern initial BCI implantations if patients are to realize the potential benefits.

## Introduction

Brain–computer interfaces (BCIs) can be categorized as stimulating or recording systems.^1^ Recording BCIs are systems that detect cortical activity and, through data extraction and algorithmic analysis, cause an action from an effector.^2^ In 1969, Fetz et al.^3^ showed that the cortical neural activity of rhesus macaques could be detected and used to trigger the dispensing of food. Since this landmark study, interest in BCIs developed rapidly throughout the remainder of the twentieth century, in tandem with an exponential increase in studies relating to BCIs at the turn of the millennium as technological capability aligned with theoretic knowledge.^2^^,^^4^

BCIs may be invasive (implanted directly onto the brain, as in electrocorticography or intracortical arrays) or noninvasive (placed on the scalp, such as in electroencephalogram hardware).^2^ In recent years, BCI development has rapidly progressed along with practical applications. Examples of such applications include using BCIs to operate word spelling systems,^5^^,^^6^ control computer programs,^7^^,^^8^ move robotic prostheses,^9^^,^^10^ control wheelchairs,^11^ or assist with neurorehabilitation.^12^ Simultaneous to the promise that BCIs offer in health care, ethical concerns remain.^13^ Current proposed applications of BCIs are predominantly restorative, but in the future, BCIs may have the potential to augment function of otherwise healthy patients,^1^ such as enhancing human memory,^14^^,^^15^ and creating brain-to-brain communication systems.^1^^,^^16^ In addition, invasive BCI implantation is associated with surgical risks including bleeding and infection^2^ or damage to eloquent brain tissue,^17^ which need to be considered when discussing the overall paradigm of BCIs. Neurosurgery has a rich history of driving the inception and development of novel technologies,^18^ and as BCI research evolves, neurosurgeons and the neurosurgical team will be responsible for implantation of BCI systems.^1^ The neurosurgical team will have a vital role in the evolving paradigm of BCI, both technologically and ethically, and are therefore suitably placed to discuss the risk associated with this disruptive technology.^19^

To our knowledge, there has been limited research into the acceptability of BCI to the neurosurgical team. We aimed to establish the neurosurgical team's baseline knowledge of BCIs and identify attitudes toward different applications of invasive BCI. This process includes assessment of willingness to participate in the insertion of different applications of invasive BCI and determining perceived appropriateness of different invasive BCI applications.

## Methods

### Overview of Methods

**Figure 1:**
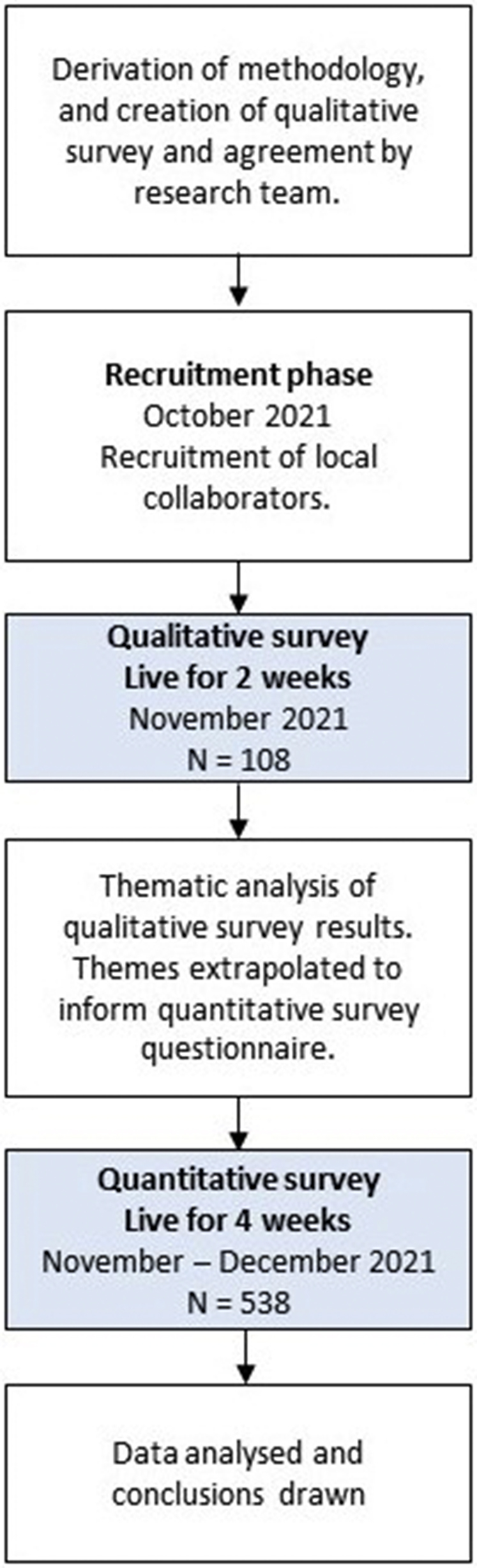
Study methodology flow diagram.

A cross-sectional, 2-stage, mixed-method (qualitative and quantitative) survey was performed in keeping with the precedence within the literature (Figure 1).^20^^,^^21^ The first-stage qualitative survey (Table 1) assessed baseline understanding and attitudes of the neurosurgical team toward BCIs. Thematic analysis led to the emergence of themes, used to generate the second-stage quantitative survey (Table 2), which presented scenarios and assessed participant acceptability of proposed BCI implementation. Survey methodology and distribution adhered to recommended practice^22^ and this survey has been reported in accordance with CROSS (Consensus-Based Checklist for Reporting of Survey Studies).^23^ Ethical approval was not required for this study because no patient or clinical data were collected, and the study was performed to plan and advise on future research.^24^

**Table 1:** Qualitative Survey Blank Space Questions

Q1	Have you encountered brain–computer interfaces (BCIs) before, and if so, in what context? (Clinical practice, scientific literature, newspapers and magazines etc.)
Q2	What is your current understanding of BCIs?
Short explanation on BCIs (see Supplementary Table 1)	
Q3	What do you think the main advantages of intracranial BCIs in neurosurgery may be? Can you give any examples?
Q4	Would you have any concerns if your team was asked to insert an invasive BCI? If yes, what would they be?

BCI, brain–computer interface.

**Table 2:** Quantitative Survey Case Vignettes

Case 1:	A patient undergoes a decompressive craniectomy following a malignant cerebral infarct and is now unable to verbally communicate. The patient is scheduled for cranioplasty (replacement of the bony defect with a titanium plate). An invasive BCI is planned to be inserted during the same operation. The invasive BCI will detect neural signals and help the patient communicate through “thought-to-speech”
Case 2:	A patient who has suffered a stroke is unable to verbally communicate. An invasive BCI is planned for insertion to help this patient communicate. Electrode grids will be placed over the patient's cerebral cortex which detect neural signals associated with speech, which will then be decoded to generate an audio output, in a “thought-to-speech” mechanism. Insertion will require a general anesthetic and drilling through the skull to access the brain
Case 3:	An amputee plans to have an invasive BCI inserted to assist in control of a prosthesis. Neural signals corresponding to the specific desired movements will be detected and interpreted, resulting in coordinated movement of a forearm and hand prosthesis. Insertion will require a general anesthetic and drilling through the skull to access the brain
Case 4:	A healthy individual is planned to have a commercial invasive BCI fitted that enables them to control and interact with computer software using neural activity. Insertion will require a general anesthetic and drilling through the skull to access the brain
Case 5:	A social media company develops an invasive BCI which better enables users to access and interact with numerous software, including enhanced interaction with online games, social media, and virtual reality environments. Insertion will require a general anesthetic and drilling through the skull to access the brain
Case 6:	An invasive BCI is developed to enable military personnel to communicate with one-another without verbalizing speech. Insertion requires general anesthetic and drilling through the skull to access the brain

BCI, brain–computer interface.

### Participants

Participants were invited to participate internationally and included members of the neurosurgical team who would be directly involved in the surgical implantation of invasive BCIs. The neurosurgical team included neurosurgeons, anesthetists, and operating room nurses.^20^ Trainee and consultant grades were included, whereas student and nontraining roles were excluded.

### Distribution

Surveys were created (GoogleForms [Google, Mountain View, California, USA]) and distributed via a network of international collaborators. Collaborators were provided with study information sheets and sought responses from their individual units. Collaborator status was achieved if local recruits were able to collect 23 responses in total, with guidance advising to collect 3 responses for the qualitative survey, and 20 responses for the quantitative survey. The qualitative survey was distributed exclusively via local collaborators. To encourage participation, the quantitative survey was distributed both via local collaborators and via social media (Twitter and Facebook). The qualitative survey was live for 2 weeks (November 2021), and the quantitative survey live for 4 weeks (November and December 2021). Data for both surveys were collected independently as 2 individual cluster sampling surveys.

### First-Stage Qualitative Survey

Participant demographics and occupation were collected, followed by 2 open questions regarding their understanding of BCIs. An introduction to BCI was provided, followed by 2 further open questions relating to the perceived advantages and concerns of BCIs (Table 1; Supplementary Table 1). Answers were thematically analyzed and coded to identify core themes, which were used to guide the questions and scenarios posed in the second-stage quantitative survey.

### Second-Stage Quantitative Survey

Demographics including age, gender, country of residence, and occupation were recorded. Current understanding of BCIs was recorded before a series of 6 case scenarios regarding intracranial BCIs (Table 2; Supplementary Table 2). For each case vignette, participants were asked 2 questions: “Do you agree or disagree that this is an appropriate use of BCI?” and “Would you be happy to be involved as a member of the surgical team in this example of a BCI?” Answers were recorded using a 5-point Likert scale (1, strongly disagree; 2, disagree; 3, neither agree nor disagree; 4, agree; and 5, strongly agree; identical ranking for baseline understanding response). The Likert scale was designed in accordance with existing recommendations.^25^

### Data Analysis

Qualitative survey data were thematically analyzed to identify themes, in accordance with existing guidance on thematic analysis.^26^ Free text answers were screened for themes, and coded to facilitate data analysis. Data from the quantitative survey were analyzed using GraphPadPrism5 software (GraphPad Software Inc., San Diego, California, USA). Data from all respondents were analyzed, followed by subgroup analysis for occupation and age. Inferential statistics for quantitative data were conducted in accordance with accepted statistical theory.^27^ Our quantitative data were discrete, ordinal data and values were assigned a numeric rank (1, strongly disagree; 2, disagree; 3, neither agree nor disagree; 4, agree; and 5, strongly agree). Median response was reported as a measure of central tendency. Statistical analysis of responses between 2 datasets used Mann-Whitney *U*, whereas analysis among ≥3 groups used a Kruskal-Wallis test, with a *P* value <0.05 denoting statistical significance. Where a Kruskal-Wallis test was used, a Dunn multiple comparison test was subsequently used to compare individual groups. Quantitative data are also presented using descriptive statistics.

## Results

### First-Stage Qualitative Survey

A total of 108 responses were collected from participants in 23 countries (Figure 1). Most respondents were male (79/108; 73%). The modal age group was 30–39 years (47/108; 44%), followed by 20–29 years (31/108; 29%), 40–49 (16/108; 15%), 50–59 years (6/108; 6%), and ≥60 years (3/108; 3%). Occupation was reported as neurosurgeon (43/108; 40%), anesthetist (39/108; 36%), and operating room nurse (26/108; 24%). BCIs had been encountered in a range of settings by other respondents, including in scientific literature (31/108; 29%); media including social media, television, and the Internet (20/108; 19%); and clinical practice (6/108; 6%). Half of respondents (54/108; 50%) had not encountered BCIs before in any context.

Participants were asked about their understanding of BCIs (Table 1; Supplementary Table 1) and 56% reported a poor baseline knowledge of BCI. The responses were coded into 4 main themes: 1) basic understanding; 2) potential applications; 3) implications for neurosurgery; and 4) emotional reaction to BCI.

Respondents were able to identify that BCIs “…allow their users to communicate or control external devices” and that BCIs used “signals from the brain.” No participants were able to describe in detail the mechanisms of BCI data extraction or command output. Respondents noted numerous potential applications of BCI, including controlling “…muscle groups, prosthetics, or an external thing like a cursor [to] allow users to communicate or control external devices.” Several respondents also highlighted the potential for BCIs to be used in certain disease targets, such as in neurodegenerative disease, paralysis, or in amputees. A few respondents mentioned that BCIs may improve the safety of neurosurgical operations, focusing on the use of BCI from the surgeon's perspective rather than that of the patient. One participant noted the wide-ranging effects that BCI may have on neurosurgery, writing “…a new era of human future life minimising disability and improving life quality.” Numerous participants showed positive emotive reactions including listing BCIs as “good,” a “great thing,” a “highly promising field,” and technology that “can help hugely.”

Participants were asked about the anticipated advantages of BCIs (Table 1; Supplementary Table 1). Many named at least one proposed benefit of BCIs (95/108; 88%). The most common theme of BCI advantages was disease targets, including spinal cord injury, locked-in syndrome, and traumatic brain injury. Other reported uses included restorative movement, use of prostheses, restoration of special senses, and promotion of neuroplasticity and rehabilitation. Another theme for BCI advantages was intraoperative assistance, including intraoperative cortical mapping and patient assessment. Anesthetic respondents highlighted how BCIs may be used as a technological adjunct in “assessment of neural activity during or after surgery, [and] assessing anaesthetic depth.” Several participants mentioned the technical advantages that invasive BCI confers over its noninvasive counterparts, such as constant monitoring of neural activity compared with when users must wear surface electrodes. Another reported advantage of BCI was the impact on quality of life; 1 respondent commented that BCI may “…support users with disabilities in everyday and professional life, and increase collaboration in building their communities,” and others mentioned the significant impact that BCI may have on “improvement in quality of life…particularly around regaining independence.”

More than half of respondents (58/108; 54%) expressed concerns when questioned in the final open question about insertion of a BCI system (Table 1; Supplementary Table 1). Thematic analysis of potential concerns derived 4 themes: 1) short-term complications; 2) long-term complications; 3) lack of experience with BCI technology; and 4) ethics, security, and other concerns. Respondents frequently mentioned immediate intraoperative and postoperative complications such as infection, bleeding, seizures, delayed recovery, and the effect of BCI implantation on anesthetics. Long-term complications reported included the effect of BCI implants on brain tissue, migration and longevity of BCI implants, and the potential need for reoperation. Central to the complications was the subjective inexperience with such a novel technology and a lack of surgical experience inserting BCIs. One respondent summarized many responses by writing “…because its [BCIs] theoretical and not widely tested…we still don”t know what complications could arise; in the short-term or long-term, neurologically.” Other concerns related to the hype around BCIs, noting that they may not live up to their suggested promise, or have little clinical impact. Ethical concerns related to data security such as the hacking of hardware and access to personal data, which patient groups would be permitted BCI, and who would make that decision. Almost half of respondents (50/108; 46%) did not highlight any concerns.

### Second-Stage Quantitative Survey

**Figure 2:**
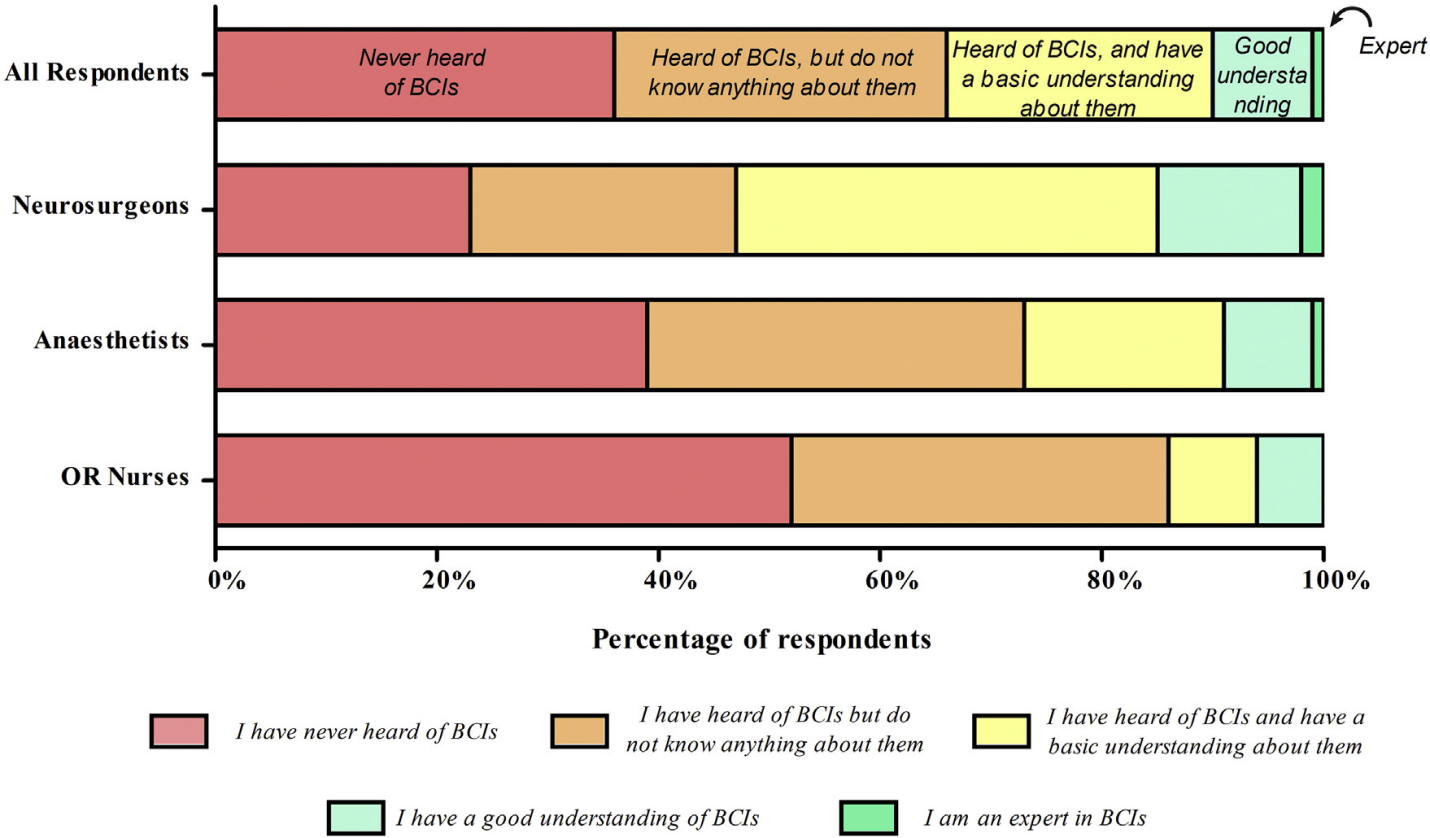
Baseline understanding of brain–computer interfaces (BCIs) among the neurosurgical team: “How would you rate your current understand of brain–computer interfaces?” OR, operating room.

A total of 538 responses from 32 different countries were obtained (Figure 1; Table 3). Most respondents were male (334/538; 62%), with the modal age group being 30–39 years (200/538; 37%). Baseline understanding of BCI from the 538 respondents was poor (Figure 2). The proportion of respondents with a good or expert understanding of BCIs was low among all specialties: neurosurgeons (15%), anesthetists (9%), and operating room nurses (6%). The difference in baseline knowledge of BCIs among specialty groups was found to be statistically significant (*P* < 0.0001, Kruskal-Wallis test), with neurosurgeons having a statistically significantly greater rank than did anesthetists (Dunn multiple comparison test, *P* < 0.05), who in turn had a greater rank than did operating room nurses (Dunn multiple comparison test, *P* < 0.05).

**Table 3:** Quantitative Survey Baseline Characteristics

Characteristic	n (%) (N = 538)
Gender	
Male	334 (62)
Female	192 (36)
Prefer not to say	7 (1)
Nonbinary	4 (1)
Occupation	
Neurosurgeon	237 (44)
Anesthetist	153 (28)
Operating room nurse	148 (28)
Age group	
<20 years	12 (2)
20–29 years	157 (29)
30–39 years	200 (37)
40–49 years	113 (21)
50–59 years	39 (7)
≥60 years	17 (3)
Response by continent	
Asia	224 (42)
Europe	182 (34)
Africa	94 (17)
North America	35 (7)
South America	3 (1)

The second-stage quantitative survey posed 6 different scenarios detailing different applications of invasive BCI (Table 2). For each scenario, participants answered 2 questions: “Do you agree or disagree that this is an appropriate use of BCI?,” and “Would you be happy to be involved as a member of the surgical team in this example of a BCI?” (Supplementary Table 1). Scenarios 1–3 related to rehabilitative applications of BCI in patients with a deficit. Scenarios 4–6 related to augmentative BCI applications in healthy individuals.

**Figure 3:**
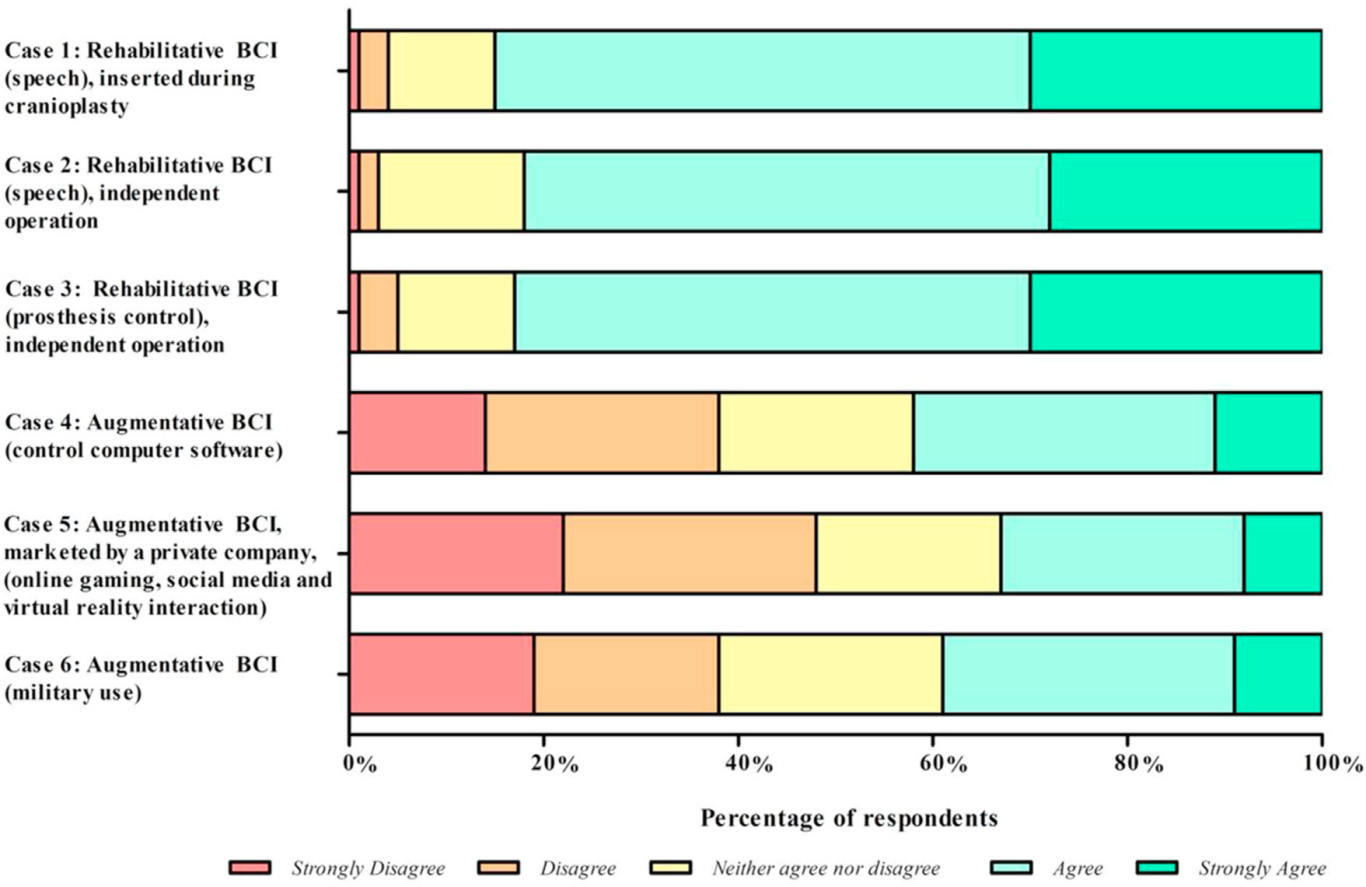
Responses of participants to the question “Do you agree or disagree that this is an appropriate use of BCI?” for brain–computer interface (BCI) case vignettes.

The respondents were largely in agreement for BCIs relating to rehabilitative purposes (83% agree or strongly agree), compared with a BCI for the augmentative application in healthy individuals (38% agree or strongly agree) (Figure 3). Although there was overall agreement for the rehabilitative BCI applications (scenarios 1–3), there remained a few (4%) of the neurosurgical team who strongly disagreed or disagreed with this application (Figure 3). Further, the augmentative applications of BCI evoked a higher percentage of strongly disagree and disagree for an augmentative BCI to control computer software (scenario 4; 38%), a BCI developed by a private social media company (scenario 5; 48%), and a BCI developed for military use (scenario 6; 38%).

**Figure 4:**
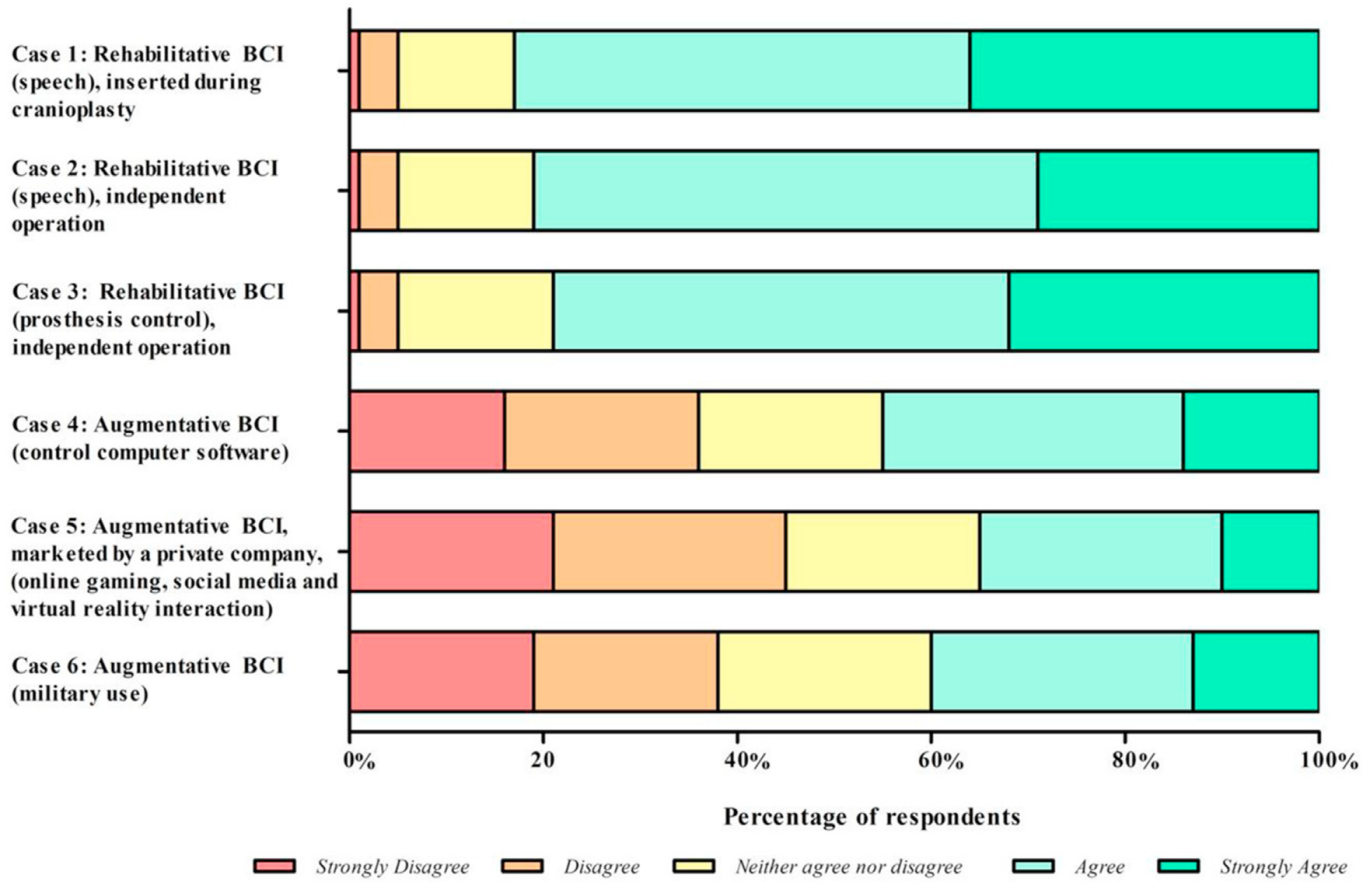
Responses of participants to the questions “Would you be happy to be involved as a member of the surgical team in this example of a BCI?” for brain–computer interface (BCI) case vignettes.

The degree to which members of the neurosurgical team were happy to place an implant mirrors the agreeability for the intended application of the discussed BCI (Figure 4). Respondents were happier to be involved with the neurosurgical team for rehabilitative BCI implantation (scenarios 1–3) than for augmentative BCI implantation (scenarios 4–6). For example, 84% of respondents agreed or strongly agreed that they would be happy to insert the BCI to assist with speech after a stroke as part of a cranioplasty operation, compared with 45% of respondents who disagreed or strongly disagreed to be a part of the team inserting an augmentative BCI developed by a private social media company (Figure 4).

**Figure 5:**
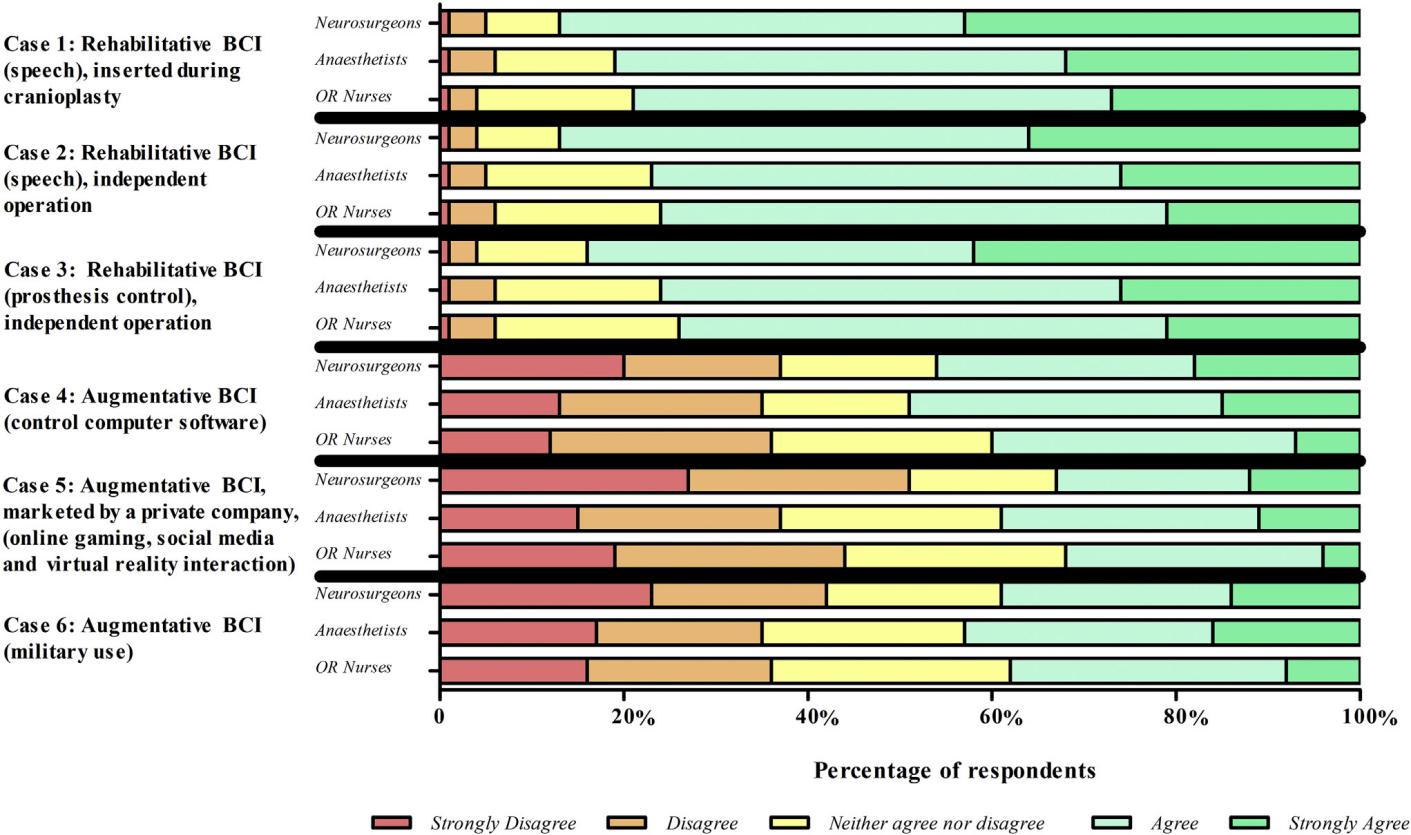
Responses by specialty to the question “Would you be happy to be involved as a member of the surgical team in this example of a BCI?” for brain–computer interface (BCI) case vignettes. OR, operating room.

Willingness of the neurosurgical team to participate in BCI insertion differed by specialty (Figure 5). Neurosurgeons were happier to insert rehabilitative BCIs than were anesthetists and operating room nurses, as shown by the highest proportion of strongly agree or agree across scenarios 1–3 (Figure 5). For these scenarios, neurosurgeons responded with an average of 86% strongly agree or agree to involvement as a member of the neurosurgical team, compared with 78% of anesthetists and 77% of operating room nurses. Anesthetists were more willing to be involved in insertion of augmentative BCIs (average 44% strongly agree or agree) than were neurosurgeons (39%) and operating room nurses (36%).

**Supplementary Figure 1:**
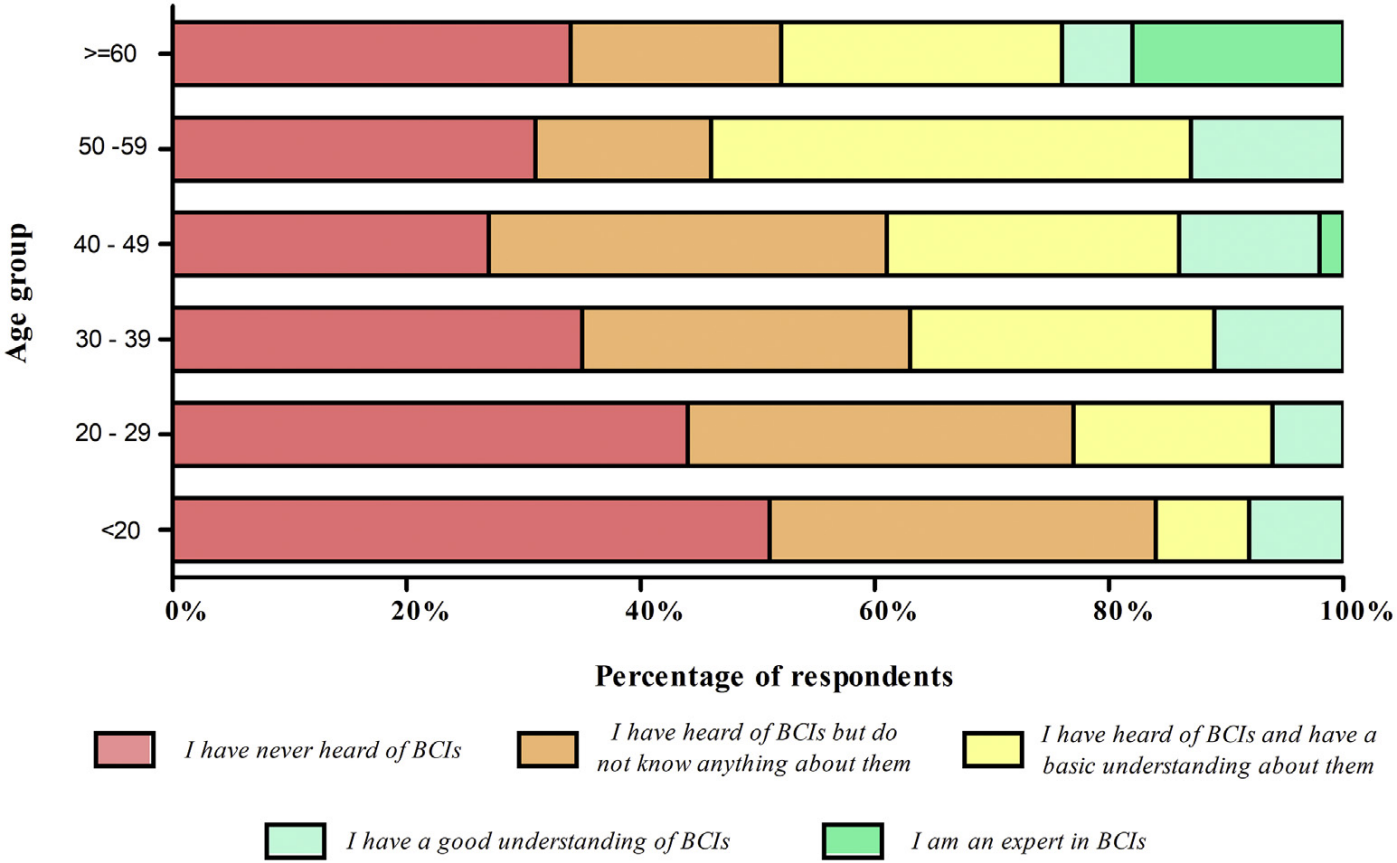
Baseline understanding of brain–computer interfaces (BCIs) among the neurosurgical team by age group: “How would you rate your current understanding of brain–computer interfaces?”

**Supplementary Figure 2:**
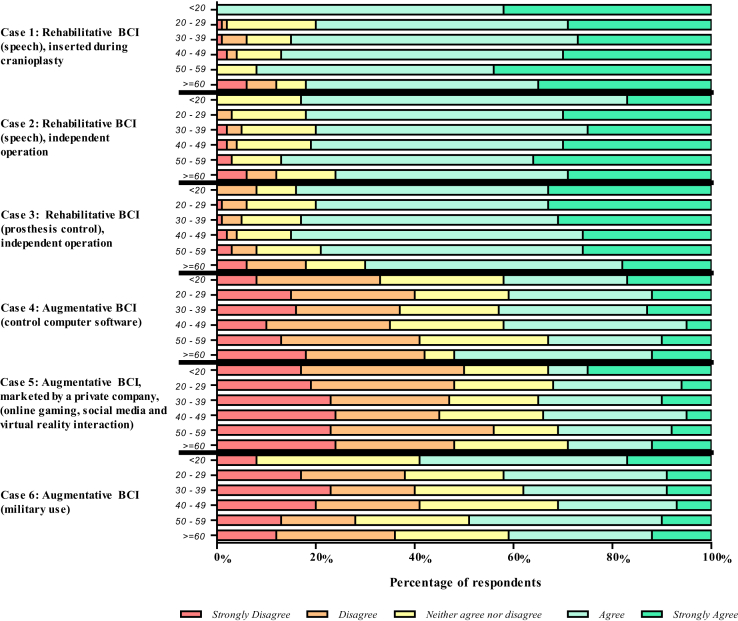
Responses by age group of participants to the question “Do you agree or disagree that this is an appropriate use of BCI?” for brain–computer interface (BCI) case vignettes.

Subgroup analysis by age group did not show any significant trends. Respondents from older age groups were more likely to have heard of BCIs, with 50% of those aged <20 years, and 43% of those aged 20–29 years reporting never having heard of BCIs, compared with 35% of ≥60-year -olds, and 31% of those aged 50–59 years (Supplementary Figure 1). Age did not significantly affect perception of appropriateness of BCI application (Supplementary Figure 2) or willingness to participate in surgical insertion.

**Supplementary Figure 3:**
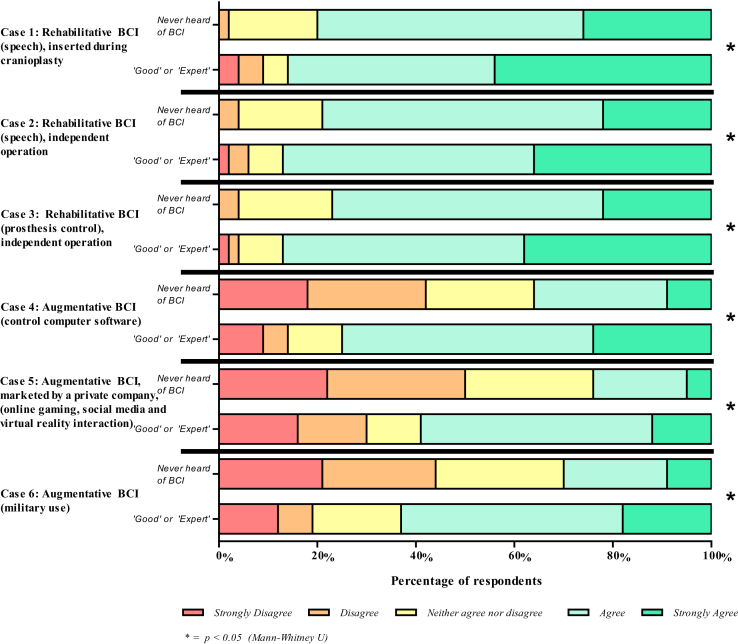
Responses by baseline knowledge of brain–computer interfaces (BCIs) to the questions “Would you be happy to be involved as a member of the surgical team in this example of a BCI?” for case vignettes.∗*P* < 0.05, Mann-Whitney *U*.

Subgroup analysis by reported level of BCI understanding showed that individuals who reported having a good or expert understanding of BCIs were statistically significantly more willing to participate in BCI insertion compared with those who had never heard of BCIs (*P* < 0.05, Mann-Whitney *U*) (Supplementary Figure 3). This significant difference was true for all cases.

## Discussion

### Key Findings

To our knowledge, this study presents the most comprehensive cross-sectional analysis of international neurosurgical teams' attitudes toward BCIs to date, with >600 participants from 32 countries. Our 2-stage survey elicited qualitative viewpoints regarding invasive BCIs, followed by a quantitative analysis of baseline knowledge, assessment of BCI applications, and willingness to participate in implantation of BCIs.

A key finding from the first-stage qualitative survey was the limited baseline understanding of BCIs among the neurosurgical team. Only 10% of neurosurgeons had a subjective good or expert understanding of BCIs. However, respondents were generally aware of the potential benefits such as the positive impact on disease targets, including spinal cord injury, locked-in syndrome, traumatic brain injury, and rehabilitative medicine. Further, respondents believed that BCIs had the potential to affect psychosocial aspects of patients' lives and empower such patients to engage meaningfully in society. Respondents reported their concerns relating to the short-term and long-term complications, their lack of experience with BCI technology including surgical technique, and the ethics and data security of BCIs.

The second-stage quantitative survey presented 6 scenarios describing rehabilitative and augmentative applications of invasive BCIs to the neurosurgical team. Respondents ranked the appropriateness of the BCI application and their willingness to be a part of the team inserting such BCIs. Scenarios were derived from real-world and anticipated examples of BCI technology, including speech assistance,^28^, ^29^, ^30^ prosthesis control,^9^^,^^31^ control of computer software, gaming and social media use,^32^^,^^33^ and military use.^34^ Respondents were agreeable to rehabilitative BCI applications, such as for speech generation and prosthesis control, and this was reflected in their willingness to be part of the surgical team inserting such BCIs (>80% for each role). However, respondents were less agreeable to augmentative BCI applications, such as military use and private social media companies. The data suggest that the neurosurgical teams' concern about being involved in BCI implantation correlates primarily with their ethical stance on the morality of the procedure. Furthermore, our data suggest that limited knowledge of BCIs correlates with unwillingness to participate in BCI insertion.

BCIs are a novel technology that divide the opinion of clinicians. Even the least controversial BCI applications have a significant minority disagreeing with their applicability, such as the rehabilitative use of a BCI to aid speech restoration after a stroke. Scoping clinician acceptability for BCI application is essential, as private industry battles to advance the BCI market.^1^ However, clinicians must engage with the decision-making process, because clinician input has been limited. For example, the Asilomar Survey^35^ surveyed 145 BCI researchers, of whom only a few were clinicians. Further, recent literature detailing the IDEAL-D (Idea, Development, Exploration, Assessment, Long-term study–Device) framework for device innovation states that device perspectives, patient perspectives, and systems perspectives, in addition to the clinician perspective, must be considered.^36^ Further work must address the ethical considerations of BCI technology and will require international collaboration to undertake patient public involvement involving legislators, social scientists, and medical ethicists; indeed, society as a whole. Similarly, regulatory legislation must keep up with the speed of development. The U.S. Food and Drug Administration issued nonbinding, regulatory guidance for implantable BCIs to help accelerate medical uses of the technology.^37^ This is the first example of a regulatory agency focusing explicitly on BCIs; however, regulation regarding augmentative BCIs is crucially missing. Future guidelines and regulations must also consider the ethical approaches to novel device innovation to enable safe advancement and to provide a regulatory environment that encourages innovation and drives forward BCI technology.^38^^,^^39^

### Comparison with the Literature

There have been few studies examining clinician acceptability of BCIs, and none specifically examining invasive BCI. Letourneau et al.^40^ give the most detailed account of clinician views to date, in their cross-sectional survey of 137 physicians directly caring for patients with severe neurologic disability in Canada, assessing clinician knowledge and potential impact of BCIs. Among their findings was a general lack of knowledge regarding BCIs, coupled with prediction from participants that BCIs stand to positively affect many patients. Nijboer et al.^35^ reported results from their Asilomar Survey in 2011, a qualitative survey conducted at an international BCI conference in the United States, drawing on views from a wide range of the multidisciplinary team, including Ph.D. students, computer scientists, neuroscientists, and engineers (a few respondents were clinicians). Grubler et al.^41^ surveyed BCI professionals, including researchers, patients, and 3 clinicians, and identified themes such as concerns regarding consent and data breaches, high expectations of BCIs, and concerns about the use of BCI in nonmedical contexts. Although such studies provide valuable insight, they do not dissect the views of the core team responsible for the surgical implantation of BCIs. Other studies have not focused on clinicians.^35^^,^^42^^,^^43^

The tariff of disease states that BCIs stand to positively affect is largely unknown, with real-world applications limited to only a few practical applications. Letourneau et al.^40^ aimed to calculate the scope of disease states that may benefit from BCI. Their team focused on a predefined set of diseases to calculate potential impact.^40^ Based on their criteria, an estimated 13,000–32,000 individuals in Canada stand to benefit from BCIs (approximately 3.6–8.9 per 10,000 when extrapolated to their population).^40^ However, although this a priori assessment of disease targets may undoubtedly benefit from invasive BCI, numerous potential applications may have gone undetected. These ‘unknown unknowns’ mean that the scope of BCI applicability may be undervalued. Furthermore, as the investigators note, their estimates for prevalence of certain neurologic disease targets was below nationwide prevalence reports, again suggesting that the impact of BCI may be greater than expected.^40^ As previously discussed, the neurosurgical team are uniquely placed to evaluate the risk/benefit ratio of BCI insertion, given their frontline experience of complications. These findings are key in the clinical translation of BCI technology; research has shown that clinician acceptability plays a significant role in the clinical impact of a technology and has wider implications for the direction of research.^44^^,^^45^

### Strengths and Limitations

This survey is the largest exploration of the neurosurgical team toward BCI acceptability. It adheres to robust methodology following precedence from the literature.^20^^,^^21^ We also consider the multidisciplinary team of neurosurgeons, anesthetists, and operating room staff, who will be responsible for the implantation of BCI.

This study has several limitations. Both qualitative and quantitative surveys were distributed in English, which may result in selection bias and makes true international review unattainable. Similarly, geographic response rate was not proportionate across continents, with a marked predominance of responses from Asia (42%) and Europe (34%). Although sampling of respondents was random, those with an interest in BCI may have been more likely to complete the survey, resulting in responder bias. However, our findings were largely consistent with existing data on BCI acceptability, which suggests a certain degree of external validity.

## Conclusions

To our knowledge, this is the most comprehensive assessment of the neurosurgical team relating to invasive BCIs. The neurosurgical team has limited baseline understanding of BCIs but are aware of the potential benefits. The neurosurgical team were agreeable to rehabilitative applications of BCI. Augmentative BCI applications remain more controversial than rehabilitative applications, yet our data highlight that many of the neurosurgical team are open to augmentative BCI. The range of views on which BCI use cases were appropriate highlights the urgent need for stakeholder consultation to guide BCI implantations in their infancy. Government, regulators, and professional bodies should engage with patient groups and the public to draft regulation and guidelines to govern BCI implantation as it moves forward.

## CRediT authorship contribution statement

**Simon C. Williams:** Data curation, Formal analysis, Methodology, Writing – original draft, Writing – review & editing. **Hugo Layard Horsfall:** Formal analysis, Methodology, Writing – review & editing. **Jonathan P. Funnell:** Writing – review & editing. **John G. Hanrahan:** Writing – review & editing. **Andreas T. Schaefer:** Supervision, Writing – review & editing**. Danyal Z. Khan**: Writing – review & editing. **William Muirhead:** Conceptualization, methodology, Writing – review & editing, Supervision. **Hani J. Marcus:** Conceptualization, methodology, Writing – review & editing, Supervision.

## Figures and Tables

**Supplementary Table 1:** Qualitative Survey

Q0:	Which collaborator asked you to fill in this survey?
Q1:	Occupation/Role: (Neurosurgeon, Anesthetist, Operating Room Nurse)
Q2:	Country of residence
Q3:	Age
Q4:	Gender (M/F/non-binary/prefer not to say)
Q	Email address ∗∗ you may choose to leave this blank ∗∗
Q5:	Have you encountered brain–computer interfaces (BCIs) before, and if so, in what context? (Clinical practice, scientific literature, newspapers and magazines etc.)
Q6:	What is your current understanding of brain–computer interfaces (BCI)?
Summary of BCIs	**Below is a short summary of brain–computer interfaces to read before completing the next section** BCIs are a technology that detect signals from the brain, process these signals, and convert them into an output. There are several key steps to how BCIs work. Firstly, signals in the brain must be detected. Signal acquisition can occur via electrodes implanted directly onto the brain (such as microelectrode arrays or subdural grids), or through electrodes planted onto the cranium (such as in EEG). The types of signals detected vary, but are often signals under voluntary control of the patient. Commonly, BCI devices record signals from the motor cortex, such that the patient can voluntarily stimulate the BCI 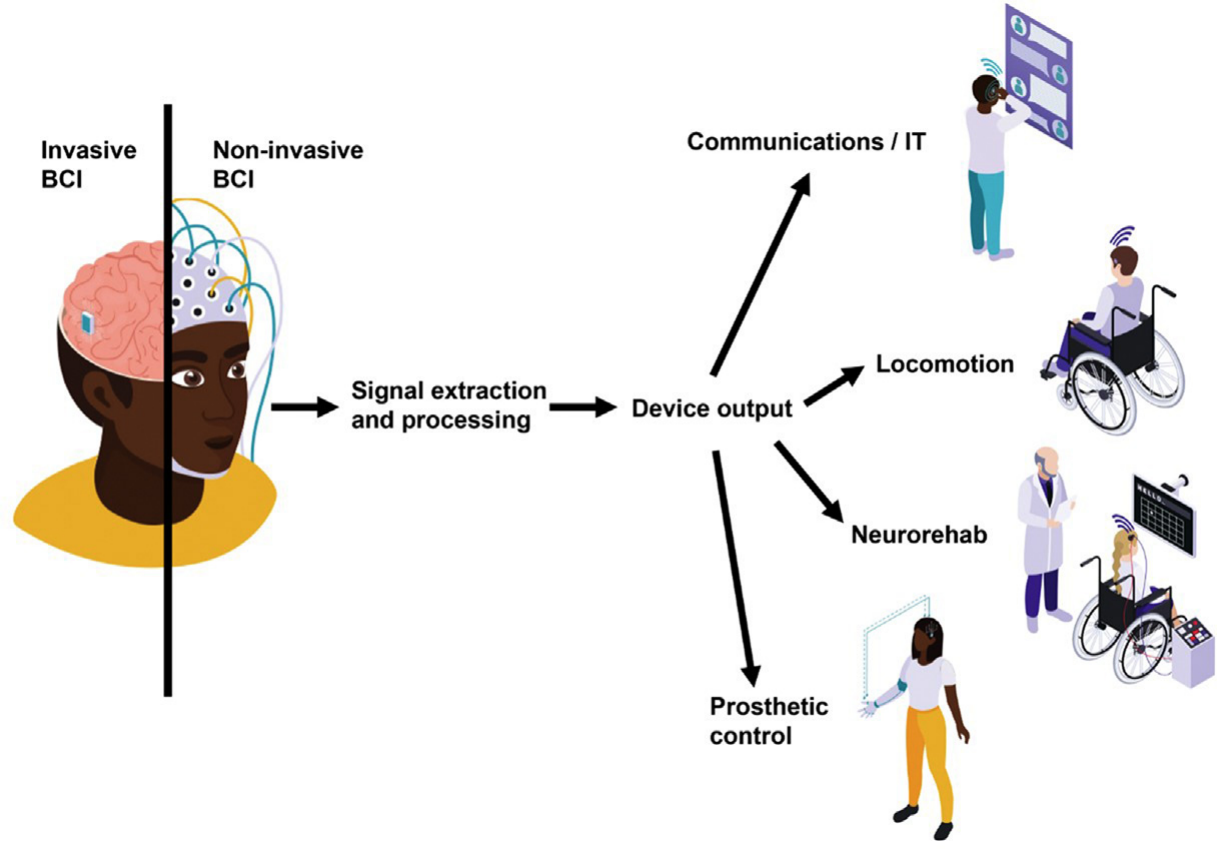 After signal acquisition, the signals are processed by a computer, which then generates an output. This output may be many different applications. In the past, BCIs have been made which help type out words on a computer screen, move a mouse cursor, move wheelchairs, and move a robotic prosthesis BCIs have the potential to be used in many areas of medicine and surgery. As described above, many uses may help patients with neurological disability in regaining function. BCIs may also help in neurorehabilitation, as increased firing in damaged areas of the brain may promote neuroplasticity Cranial surface based BCIs do not require surgical implantation. Intracranial BCIs do require surgical implantation, and would likely require a hospital admission and anesthetic. At present, real-world uses of BCIs are limited, though there are numerous possible applications. Along with healthcare, BCIs could also be used in gaming settings, and to enhance functions in well patients Example of BCI #1: Patient 1 has suffered an intraoperative stroke and now has a dense left sided hemiparesis. How would a BCI work in Patient 1? EEG electrodes could be planted onto her scalp to detect cortical activity in her motor cortex. Whenever Patient 1 now thinks about moving her arm, this activity is detected. This activity could then be linked into a computer, which decodes these signals, and triggers a flash of “well done!” on a computer screen whenever they are detected. Patient 1 now has a goal to aim for, and will actively try to trigger these signals, leading to greater activation of her motor cortex, theoretically promoting neuroplasticity and repair Example of BCI #2: Patient 2 is an upper limb amputee. The patient undergoes an operation to insert an intracranial BCI over his motor cortex, which is linked to a prosthetic arm. Neural activity is detected and corresponds to movement groups of the prosthetic, such that when the patient thinks about flexing his wrist, the prosthetic wrist flexes, and so forth
Q7:	What do you think the main advantages of intracranial BCIs in neurosurgery may be? Can you give any examples?
Q8:	Would you have any concerns if your team was asked to insert an invasive BCI? If yes, what would they be?

**Supplementary Table 2:** Quantitative Survey

Q0:	Which collaborator asked you to fill in this survey?
Q1:	Occupation/Role: (Neurosurgeon, Anesthetist, Operating Room Nurse)
Q2:	Country of residence
Q3:	Age group: (<20, 20–29, 30–39, 40–49, 50–59, ≥60)
Q4:	Gender (M/F/prefer not to say)
Q5:	How would you rate your current understanding of BCIs? I have never heard of BCIs I have heard of BCIs but do not know anything about them I have heard of BCIs and have a basic understanding about them I have a good understanding of BCIs I am an expert in BCIs
Summary of BCIs	As In Supplementary Table 1
Scenarios. For each scenario, participants are asked: • “Do you agree or disagree that this is an appropriate use of BCI?” • “Would you be happy to be involved as a member of the surgical team in this example of a BCI?” (Strongly agree, Agree, Neither agree nor disagree, Disagree, Strongly Disagree)	
Scenario 1:	Invasive BCI to assist with communication in a patient who has suffered a stroke A patient undergoes a decompressive craniectomy following a malignant cerebral infarct, and is now unable to verbally communicate. The patient is scheduled for cranioplasty (replacement of the bony defect with a titanium plate). An invasive BCI is planned to be inserted during the same operation. The invasive BCI will detect neural signals and help the patient communicate through “thought-to-speech”
Scenario 2:	Invasive BCI to assist with communication in a patient who has suffered a stroke A patient who has suffered a stroke is unable to verbally communicate. An invasive BCI is planned for insertion to help this patient communicate. Electrode grids will be placed over the patient's cerebral cortex which detect neural signals associated with speech, which will then be decoded to generate an audio output, in a “thought-to-speech” mechanism. Insertion will require a general anesthetic and drilling through the skull to access the brain
Scenario 3:	Invasive BCI to control a prosthetic limb An amputee plans to have an invasive BCI inserted to assist in control of a prosthesis. Neural signals corresponding to the specific desired movements will be detected and interpreted, resulting in coordinated movement of a forearm and hand prosthesis. Insertion will require a general anesthetic and drilling through the skull to access the brain
Scenario 4:	Invasive BCI in order to augment functions in a fit and well patient A healthy individual is planned to have an invasive BCI fitted that enables them to control and interact with computer software using neural activity. Insertion will require a general anesthetic and drilling through the skull to access the brain
Scenario 5:	Invasive BCI for social/gaming purposes A social media company develops an invasive BCI which better enables users to access and interact with numerous software, including enhanced interaction with online games, social media, and virtual reality environments. Insertion will require a general anesthetic and drilling through the skull to access the brain
Scenario 6:	Invasive BCI for military enhancement An invasive BCI is developed to enable military personnel to communicate with one-another without verbalizing speech. Insertion requires general anaesthetic and drilling through the skull to access the brain
